# Noncontact excitation of multi-GHz lithium niobate electromechanical resonators

**DOI:** 10.1038/s41378-024-00771-9

**Published:** 2024-09-05

**Authors:** Danqing Wang, Jiacheng Xie, Yu Guo, Mohan Shen, Hong X. Tang

**Affiliations:** Department of Electrical Engineering, Yale University, New Haven, CT 06511 USA

**Keywords:** Electrical and electronic engineering, NEMS

## Abstract

The demand for high-performance electromechanical resonators is ever-growing across diverse applications, ranging from sensing and time-keeping to advanced communication devices. Among the electromechanical materials being explored, thin-film lithium niobate stands out due to its strong piezoelectric properties and low acoustic loss. However, in nearly all existing lithium niobate electromechanical devices, the configuration is such that the electrodes are in direct contact with the mechanical resonator. This configuration introduces an undesirable mass-loading effect, producing spurious modes and additional damping. Here, we present an electromechanical platform that mitigates this challenge by leveraging a flip-chip bonding technique to separate the electrodes from the mechanical resonator. By offloading the electrodes from the resonator, our approach yields a substantial increase in the quality factor of these resonators, paving the way for enhanced performance and reliability for their device applications.

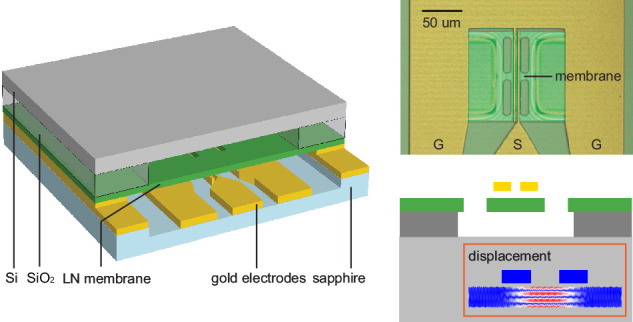

## Introduction

Electromechanical resonators are at the core of many resonator technologies, particularly in communication applications. The advent of fifth-generation (5 G) networks has increased the demand for high-performance electromechanical resonators^1^. High-frequency resonators in the upper microwave and millimeter-wave bands provide expansive bandwidths for high-data-rate communications. However, the substantial insertion loss at these frequencies causes challenges for scaling electromechanical resonators to operate within this range. Hence, high-frequency, low-loss resonators need to be devised to effectively address this challenge.

Among the various material platforms of microelectromechanical systems (MEMS), thin-film lithium niobate (TFLN) has attracted significant attention due to its excellent piezoelectric properties^2^, low acoustic loss^3^, and compatibility with large-wafer thin-film processing. Recently, strong electromechanical coupling has been reported with z-cut TFLN lamb wave modes^4^. Moreover, sub-THz TFLN electromechanical resonators have also been demonstrated^5^, highlighting the potential of this platform for broadband applications. To further enhance TFLN resonators for RF filter applications and quantum phononics research, considerable efforts have been dedicated to improving the quality factor (Q) of the devices, including reducing the surface roughness of TFLN^6^, optimizing the release process^7^, and modifying the design and materials of coupling electrodes^7–9^. However, in all these implementations, the metal electrodes are in direct contact with the lithium niobate (LN) membranes, which leads to significant mechanical losses in the electromechanical systems^10–13^.

The substantial loss accompanied by contact electrodes has motivated researchers to develop mechanical resonators excited through noncontact electrodes. Yen et al.^14^ employed capacitive-piezoelectric aluminum nitride (AlN) Lamb wave resonators to show the Q enhancement of the noncontact configuration at approximately 1 GHz. In this work, we experimentally demonstrate a noncontact TFLN electromechanical platform that can operate up to approximately 30 GHz. By employing a flip-chip bonding approach, we achieve separation of the electrodes from the resonator body through an air gap, resulting in the effective reduction of mechanical losses and suppression of spurious modes. We propose that by further reducing the gap between the electrodes and the membrane to the nanometer scale, we can still achieve a notable increase in the device quality factor while maintaining a high electromechanical coupling strength.

## Device design and fabrication

**Fig. 1:**
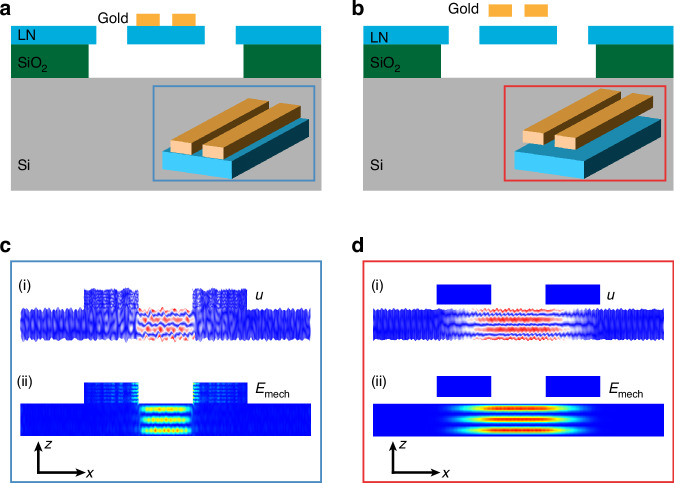
Direct-contact and noncontact resonator designs. Schematics of direct-contact (**a**) and noncontact (**b**) LN electromechanical resonators. Insets of (**a**, **b**) show three-dimensional perspectives of the central area of the electromechanical resonators. **c**, **d** Simulated on-resonance displacement field (i) and the mechanical energy distribution (ii) of the TS-3 mode. The colors from blue to red map the displacement amplitude and the stored energy from minimum to maximum

As depicted in Fig. 1, panel (a) illustrates the conventional configuration where the electrodes are in direct contact with the LN membrane, whereas panel (b) outlines our proposed scheme where an air gap is introduced between the electrodes and the membrane. In these configurations, a horizontal electric field is utilized to couple to the $z$-cut TFLN thickness-shear (TS) modes^5^ through the large ${e}_{51}$ piezoelectric coupling element. These thickness modes, bounded by the free top and bottom surfaces of the membrane, have a near-dispersionless characteristic where the mechanical resonant frequency *f*, mode order *m*, film thickness *h*, and acoustic velocity *v* are related by ${mv}=2{hf}$.

In the direct-contact system, acoustic waves inevitably propagate into the electrodes, as indicated by the displacement field *u* in Fig. 1c-i. Consequently, the electrodes provide a dissipation path for the mechanical energy ${E}_{{\rm{mech}}}$ as shown in Fig. 1c-ii. In this scenario, the mechanical damping associated with the electrodes contributes to the overall decay of the stored energy in the electromechanical resonator. In contrast, the noncontact platform circumvents this issue by fully confining the acoustic energy in the LN membrane, leveraging the significant mechanical impedance mismatch between the solid and air. This confinement is visualized through the displacement field and the energy distribution in Fig. 1d-i,-ii.

**Fig. 2:**
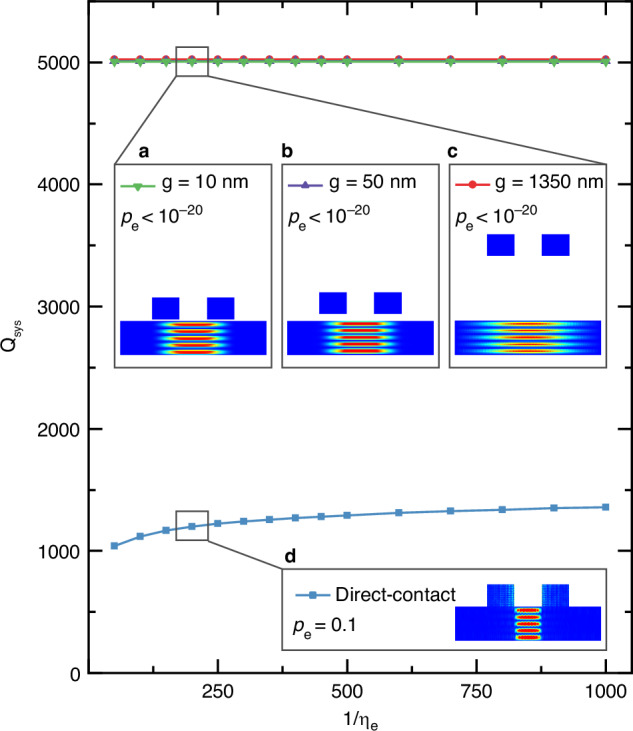
Simulated Q values for various contact configurations. Simulated system Q versus assumed gold loss factor (${\eta }_{e}$). The blue line represents the direct-contact case, whereas the green, purple, and red lines represent the noncontact cases with air gaps of 10, 50, and 1350 nm, respectively. The simulations are based on the TS-5 mode of 300 nm thick LN excited by 200 nm thick gold electrodes, and Q is fitted using the mBVD model. **a**–**d** Each configuration’s energy distribution and participation ratio at ${\eta }_{e}$ = 1/200

The loss mechanism inside an electromechanical resonator can be quantitatively described by an energy participation ratio model:

$$\frac{1}{{Q}_{{\rm{sys}}}}=\sum {p}_{i}{\eta }_{i}\approx {p}_{{\rm{m}}}{\eta }_{{\rm{m}}}+{p}_{{\rm{e}}}{\eta }_{{\rm{e}}}$$

where ${Q}_{{\rm{sys}}}$ is the mechanical quality factor of the entire system, ${\eta }_{i}$ is the loss factor^15^ of each loss channel and ${p}_{i}={E}_{i}/{E}_{{\rm{mech}}}$ is the energy participation ratio of each channel. Here, we focus on the loss induced by the LN membrane (loss factor ${\eta }_{{\rm{m}}}$), including the intrinsic damping of TFLN and the anchor loss, and the loss associated with the gold electrodes (loss factor ${\eta }_{{\rm{e}}}$). In the noncontact scenario, the mechanical energy confined in the TFLN membrane is dominant, whereas ${p}_{{\rm{e}}}$ is negligible; this contributes to a high-quality electromechanical resonator. To numerically validate the advantage of the noncontact platform, we perform finite-element method (FEM)-based simulations (COMSOL) to verify its Q-enhancement. For the damping parameters used in the simulations, we choose a realistic value of 1/5000 as ${\eta }_{{\rm{m}}}$ for the TFLN, based on the extracted data from LN acoustic delay lines^16^. Since a unified value of the gold loss factor has not been attained, we sweep across a broad range of ${\eta }_{{\rm{e}}}$ (1/50 to 1/1000^13,17,18^) to demonstrate the superiority of the noncontact platform in all these scenarios. The corresponding system Q of the fifth-order TS mode (TS-5) at 30 GHz is shown in Fig. 2, for four different configurations: (a) 10 nm air gap ($g$); (b) 50 nm air gap; (c) 1350 nm air gap; and (d) electrodes in contact with the membrane. In the direct-contact device, the gold electrodes vibrate with the LN membrane, as shown in Fig. 2d. For all loss factors ${\eta }_{{\rm{e}}}$ considered, the energy participation ratio of the electrodes (${p}_{{\rm{e}}}$) ranges from 4 to 40%, which constrains the mechanical Q of the system to values far below 5000. Moreover, the spurious modes introduced by electrodes broaden the resonant spectrum, leading to a decrease in the observed system Q. However, in the noncontact platform, as shown in Fig. 2a–c, the air gap prevents acoustic waves from propagating into the electrodes, resulting in a significantly reduced ${p}_{{\rm{e}}}$ (less than ${10}^{-20}$, obtained from simulation), and the system Q is close to the intrinsic value of the TFLN, regardless of the size of the air gap, demonstrating advantages over the direct-contact platform.

**Fig. 3:**
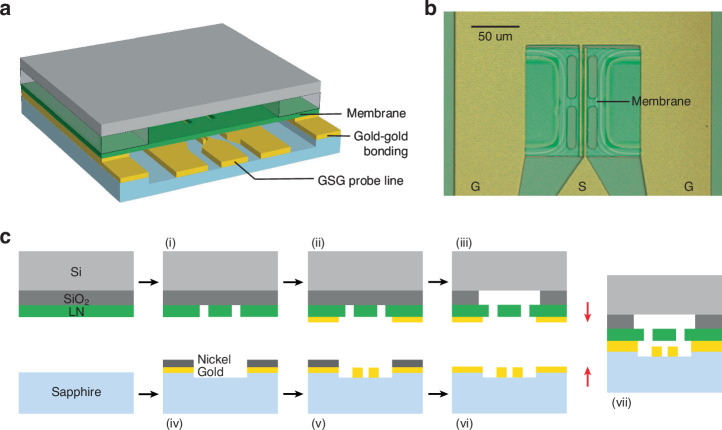
Fabrication flow and device image. **a** Schematic of a flip-chip bonded noncontact electromechanical resonator (not to scale). **b** Optical microscope image of a device, viewed from the top electrode side. The width of the membrane is 16 μm and the length is 140 μm. **c** Fabrication flow: (i) LN is etched by Ar ion milling. (ii) Gold is deposited at the bonding area. (iii) The SiO_2_ buffer layer is removed in BOE, and the chip is dried in CPD. (iv) Gold and nickel are deposited at the bonding area of the sapphire chip, and the remaining area is etched down for ~150 nm. (v) 200 nm thick gold electrodes are deposited. (vi) Nickel is removed. (vii) The two chips are bonded

We employ a flip-chip bonding technique to realize a narrow air gap between the TFLN membrane and the electrodes. The complete resonator construction is depicted in Fig. 3a, where a sapphire chip with metal electrodes is bonded to a silicon chip with suspended LN membranes using a gold-gold bonding technique^19^. The fabrication process is outlined in Fig. 3c. For the LN membrane chip, we begin with a chip comprising LN (300 nm) and SiO_2_ (5 μm) on a silicon substrate. The release windows are defined in the LN layer by patterning HSQ resist through electron-beam lithography, followed by Ar ion milling (Fig. 3c-i). A bonding gold layer is then deposited through a lift-off process (Fig. 3(c-ii)). The membrane chip is subsequently released in a buffered oxide etchant (BOE) that isotropically removes the SiO_2_ layer beneath. The released membrane chip is dried by a critical point dryer (CPD) (Fig. 3c-iii). For the electrode chip, we start with a bare sapphire substrate. We opt for sapphire due to its excellent microwave properties and transparency that aids in flip-chip alignment. Typically, the surface roughness of the bonding gold is required to be sub-nm; therefore, its thickness is controlled to be within the range of several tens of nm to achieve the best bonding result. In this step, we deposit 40 nm thick gold as the bonding layer. Given that this layer is thinner than the 200 nm thick gold electrodes in our design, we compensate for the thickness difference by creating a recessed area in the sapphire substrate to accommodate the electrodes (Fig. 3c-iv). This step uses Cl_2_ and BCl_3_ plasma etching, with nickel as the hard mask. Then, gold electrodes are deposited using a lift-off technique with Copolymer/PMMA resists (Fig. 3c-v). The nickel mask is finally removed by Piranha solution before bonding (Fig. 3c-vi). In the final step, we use a commercial bonding tool to bond the LN membrane chip and the electrode chip, resulting in a well-defined air gap between the membrane and electrodes (Fig. 3c-vii). The optical microscope image of a completed flip-chip bonded device is shown in Fig. 3b.

## Results and discussion

A ground-signal-ground (GSG) probe is galvanically connected to the electrodes on the sapphire chip for the measurements of the suspended mechanical resonators. The electrode pads on the sapphire chip are designed to protrude beyond the edge of the TFLN, facilitating probe access, as shown in Fig. 3a. Using the piezoelectricity of LN, the mechanical mode is coupled to the microwave field, and the reflection signal can be collected by a vector network analyzer.

**Fig. 4:**
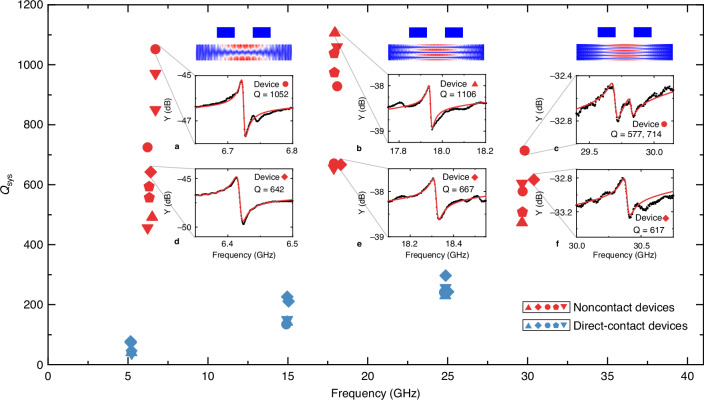
Device performance in multi-GHz range. Measured Q versus modal frequency for noncontact devices (red symbols, LN thickness ~300 nm) and direct-contact devices (blue symbols, LN thickness ~370 nm). Five devices from each category are marked as up-triangles, diamonds, circles, pentagons, and down-triangles. Insets (**a**–**f**) plot the admittance spectra near the first three odd TS modes. The raw data, presented as black dots, are fitted by the mBVD model as red curves. **a**–**c** Data from devices with the highest Q for each mode. **d**–**f** Spectra from a chosen device (red diamond)

The admittance spectra near the first three odd TS modes are plotted in the insets (a–f) of Fig. 4. A modified Butterworth-Van Dyke (mBVD) model is used to fit the experimental data^20^; these data are represented by solid lines in the insets of Fig. 4. In the mBVD model, a mechanical resonator is depicted as a combination of three lumped elements in series (${Z}_{{\rm{mech}}}=1/\left({\rm{i}}\omega {C}_{{\rm{mech}}}\right)+{\rm{i}}\omega {L}_{{\rm{mech}}}+{R}_{{\rm{mech}}}$). In addition to exciting the resonator, the coupled electrode pair is equivalent to a mutual capacitor (${C}_{{\rm{M}}}$). The mechanical Q of devices is extracted as. $\sqrt{{L}_{{\rm{mech}}}/{C}_{{\rm{mech}}}}/{R}_{{\rm{mech}}}$. The Q values of the multiple mechanical resonators excited by both noncontact and direct-contact electrodes are plotted in the main graph of Fig. 4, which demonstrates significant Q enhancement of the noncontact platform. Notably, the highest experimental Q values are 1052 for the TS-1 mode, 1106 for the TS-3 mode, and 714 for the TS-5 mode, as depicted in Fig. 4(a–c). To illustrate the systematic Q-improvement of the noncontact devices, we plot the admittance spectra of a chosen device with average performance, as shown in Fig. 4d–f. Remarkably, the Q values of the three modes, all exceeding 600, are substantially higher than those observed in the direct-contact system. Therefore, despite the variations in the measured Q values in each category, it is clear that the noncontact platform systematically achieves a higher Q performance than the direct-contact platform. Moreover, as indicated by the simulations in Fig. 2, this noncontact technique for enhancing Q is universally applicable to piezoelectric materials with various loss properties.

To estimate the achieved air gap distance, we image the membrane chip using a 3D optical profilometer and observe a buckling of approximately 1.3 μm in the suspended 300 nm thick LN film toward the silicon substrate. The extent of this buckling is consistent with the optical interference patterns observed on the noncontact device, as shown in Fig. 3b. The buckling of the membrane could be derived from the intrinsic compressive stress of the LN films and is under further investigation. Since the resulting gap distance exceeds the designed value, the electromechanical coupling coefficients (${K}^{2}$, defined as ${C}_{{\rm{mech}}}/{C}_{{\rm{M}}}$) are lower than expected, measuring 0.02% and 0.01% for the TS-3 and TS-5 modes of the device, as represented by the red diamond symbol in Fig. 4d–f, respectively. According to FEM simulations, if the air gap reaches 50 nm as designed, the ${K}^{2}$ values of the noncontact case are 2.5% and 0.9% for the TS-3 and TS-5 modes, respectively, both of which are approximately 70% of those in the direct-contact case. Consequently, addressing the buckling in the LN thin films will be a primary focus in the upcoming stages of development. The noncontact configuration with reduced buckling will deliver high performance in terms of both achievable quality factors and ${K}^{2}$ characteristics.

## Conclusions

We develop a flip-chip bonding technique to excite LN resonators via noncontact electrodes, resulting in a notable improvement in the mechanical Q with respect to the direct-contact case in the multi-GHz frequency regime (up to 30 GHz). This configuration not only effectively reduces the loss associated with the electrodes, but also has the potential to maintain a high electromechanical coefficient. The device structure demonstrated here can be extended to other piezoelectric materials, such as AlN. With further development, the fabrication process demonstrated in this work will permit the fabrication of even thinner nanomembranes below 100 nm. Due to these collective advantages, noncontact electromechanical resonators are effectively suited for high-frequency applications.
